# IL-33, IL-37, and Vitamin D Interaction Mediate Immunomodulation of Inflammation in Degenerating Cartilage †

**DOI:** 10.3390/antib10040041

**Published:** 2021-10-26

**Authors:** Vikrant Rai, Mohamed M. Radwan, Devendra K. Agrawal

**Affiliations:** Department of Translational Research, Graduate College of Biomedical Sciences, Western University of Health Sciences, Pomona, CA 91766, USA; vrai@westernu.edu (V.R.); mradwanahmed@westernu.edu (M.M.R.)

**Keywords:** cartilage degeneration, osteoarthritis, inflammation, interleukin-33, interleukin-37, vitamin D supplementation, macrophage polarization

## Abstract

Chronic joint inflammation due to increased secretion of pro-inflammatory cytokines, the accumulation of inflammatory immune cells (mainly macrophages), and vitamin D deficiency leads to cartilage degeneration and the development of osteoarthritis (OA). This study investigated the effect of vitamin D status on the expression of mediators of inflammation including interleukin (IL)-33, IL-37, IL-6, tumor necrosis factor (TNF)-α, toll-like receptors (TLRs), damage-associated molecular patterns (DAMPs), and matrix metalloproteinases (MMPs) in degenerating the cartilage of hyperlipidemic microswine. Additionally, in vitro studies with normal human chondrocytes were conducted to investigate the effect of calcitriol on the expression of IL-33, IL-37, IL-6, TNF-α, TLRs, DAMPs, and MMPs. We also studied the effects of calcitriol on macrophage polarization using THP-1 cells. The results of this study revealed that vitamin D deficiency is associated with an increased expression of IL-33, IL-37, IL-6, TNF-α, TLRs, DAMPs, and MMPs, while vitamin D supplementation is associated with a decreased expression of the former. Additionally, vitamin D deficiency is associated with increased M1, while vitamin D-supplemented microswine cartilage showed increased M2 macrophages. It was also revealed that calcitriol favors M2 macrophage polarization. Taken together, the results of this study suggest that modulating expression of IL-33, IL-6, TNF-α, TLRs, DAMPs, and MMPs with vitamin D supplementation may serve as a novel therapeutic to attenuate inflammation and cartilage degeneration in osteoarthritis.

## 1. Introduction

An imbalance between the regenerative and degenerative processes of cartilage with a predominance of degenerative factors results in cartilage damage and the development of osteoarthritis (OA). Recent studies have suggested the role of inflammation in the pathogenesis of OA; damage-associated molecular patterns (DAMPs) being instigators and macrophage, the main effectors of inflammation [1,2,3]. The role of pro-inflammatory cytokines, including IL-6, IL-1β, TNF-α, IL-33, and IL-8, secreted because of the activation of downstream pro-inflammatory signaling and anti-inflammatory cytokines, including IL-10 and IL-37, in inflammation, macrophage polarization, and osteoarthritis, has been reported [4,5,6,7,8,9,10,11,12,13,14]. These studies signify the role of inflammation in the pathogenesis of OA and the suppression of inflammation as a strategy to attenuate the progression of OA [1,4,5,6,7,8,9,10,11,12,13]. Our previous study showed the presence of the high mobility group box protein-1 (HMGB-1), the receptor for advanced glycation end products (RAGE), S100 proteins, and macrophages in the osteoarthritic human knee and hip joint cartilage [3]. An increased density of macrophages in association with the increased expression of mediators of inflammation in degenerating cartilage of vitamin D-deficient microswine and the association of vitamin D supplementation with decreased macrophages and increased triggering receptor expressed on myeloid cells (TREM)-2 expression, an anti-inflammatory receptor, have been reported previously by us [1]. These results suggest the presence of inflammation and macrophages in osteoarthritic cartilage.

Vitamin D deficiency is associated with chronic inflammation and the increased secretion of IL-33. IL-33 increases the secretion of pro-inflammatory cytokines, namely IL-4, IL-5, IL-6, IL-8, and IL-13. These cytokines and IL-33 play a crucial role in the pathogenesis of OA [14,15,16,17,18,19,20]. The association of low plasma levels of vitamin D with cartilage degeneration, joint space narrowing, development, and incidence of OA suggest a potential role of vitamin D deficiency in the pathogenesis of cartilage degeneration and OA [1,21,22,23,24,25,26]. However, there are discrepancies in the results for the association between vitamin D deficiency and OA [27,28]. These discrepancies may be due to the polymorphism of the VDR gene and its association with OA [29,30,31,32]. However, quadriceps muscle weakness, muscles, tendon, and ligaments fatty infiltration, joint weakness, and decreased weight-bearing capacity of the joint with vitamin D deficiency indicate a significant correlation of vitamin D deficiency with OA [1,2,3,33]. Moreover, chronic inflammation in obesity, a major risk factor for OA, is associated with vitamin D deficiency. Furthermore, the association of vitamin D deficiency with chronic inflammation suggests that the concurrent presence of inflammation and vitamin D deficiency may potentiate each other to have a detrimental effect on the cartilage mediating the increased degeneration of the cartilage and thereby OA. Thus, vitamin D supplementation to attenuate inflammation, fatty infiltration, and strengthen the muscles may attenuate inflammation and cartilage loss and progression of OA. Various studies implicate the role of vitamin D deficiency in the structural change and degeneration of cartilage; however, the role of vitamin D in preventing the onset and worsening of OA is debatable [1,21,22,23,25,34,35,36]. Additionally, whether IL-33-mediated inflammation in OA cartilage [14] may be attenuated with vitamin D has not been investigated. In this study, we have investigated the effects of vitamin D status on the expression of mediators of inflammation including IL-33, and the effect of calcitriol on the expression levels of IL-33, IL-6, TNF-α, TLRs, DAMPs, MMPs, and macrophage polarization with a hypothesis that vitamin D supplementation attenuates IL-33-mediated inflammation and enhances M2 macrophage polarization, thereby decreasing inflammation in the cartilage.

## 2. Materials and Methods

### 2.1. Porcine Model

This study was conducted using Yucatan female microswine [37,38]. The research protocol for the study was approved by the Institutional Animal Care and Use Committee (IACUC) at Creighton University (IACUC approval number: IACUC#831), Omaha, NE. Yucatan microswine (30–40 lbs) purchased from Sinclair Laboratories, Columbia, MO, USA, were kept in a controlled environment in the Animal Resource Facility of Creighton University, Omaha, NE. NIH standards and USDA guidelines were followed for their care and experimental protocol. Microswine were fed a pelleted high cholesterol diet (Teklad Miniswine Diet, Harlan Laboratories) with 17.4% protein, 45.2% carbohydrates, and 10.0% fat by weight. Of the total kilocalories from the diet, 20.4% of the kilocalories were from protein, 53.1% of the kilocalories were from carbohydrates and 26.4% of the kilocalories were from fat. The pelleted diet was either deficient in Vitamin D (TD-150251), sufficient (TD-150250: 1.5 IU/gD, HVO, 4% Chol, NaCh) with 1500 IU/D of vitamin D3, or supplemented (TD-150252: 5 IU/gD, HVO, 4% Chol, NaCh) with 5000 IU/D of Vitamin D3. Based on our previous experience and other studies, vitamin D3 supplementation of 1500 IU/day for sufficient and 5000 IU/ day for the supplemented group were added to achieve normal (21–29 ng/mL) and supplemented serum levels of vitamin D in microswine [39,40,41]. Serum 25(OH) D levels were measured regularly and recorded. The swine were placed into three respective categories based on the 25(OH) D levels. The 25(OH) D level parameters for the classification were as follows: vitamin D deficient (VDDef) swine were ≤20 ng/mL, vitamin D sufficient (VDSuff) swine were 30–44 ng/mL, and vitamin D-supplemented (VDSupp) swine were >44 ng/mL. Five swine were included in VDDef, 10 in VDSuff, and 5 in the VDSupp group for a total of 20 swine in this study. These swine were primarily used for cardiovascular studies, but the presence of degenerating cartilage in vitamin D-deficient swine led to our interest to investigate the mediators of inflammation and its correlation with vitamin D status in the cartilage. The swine were fed a high cholesterol diet for one year and femoral cartilage and joint fat tissues were collected after the swine were euthanized (as described in [1]).

### 2.2. Tissue Acquisition

The knee joint cartilage tissues were harvested quickly after euthanasia, transported to the laboratory, and fixed in 4% formalin for 24 h. Each specimen was transversely sectioned at 2 mm, processed in Sakura Tissue Tek VIP Tissue Processor, and embedded in paraffin. Thin sections (5 μm) were cut using a microtome (Leica, Wetzlar, Germany) and subsequently placed on slides for hematoxylin and eosin and immunofluorescence studies.

### 2.3. Immunofluorescence Studies

Deparaffinization, rehydration, and antigen retrieval were done before immunostaining. Immunofluorescence (IF) staining was done as per standard protocol in our laboratory. The tissues were incubated with rabbit anti-CD86 (ab53004) for M1a, rabbit anti-CD206 (ab64693) for M2a, rabbit anti-CD163 (ab87099) for M2b, rat anti-IL-10 (JES3-19F1) for M2c, mouse anti-CD 14 (ab182032) for macrophage, rabbit anti-IL37 (ab153889) at 1:200 dilution, rabbit anti-ST2 (06-1116) at 1:100 dilution and goat anti-TLR2 (sc-8690), rabbit anti-TLR4 (sc-10741), mouse anti-MyD88 (sc-136970), and rabbit anti-IL33 (sc-98659) at 1:50 primary antibodies overnight at 4 °C. This was followed by washing with phosphate-buffered saline (PBS) and incubation with Alexa Fluor 594 (red) and Alexa Fluor 488 (green) conjugated secondary antibodies (Invitrogen, Grand Island, NY, USA) at 1:1000 dilution for 1 h at room temperature. DAPI (4, 6-diamidino-2-phenylindole) with mounting medium was used to stain nuclei. Negative controls were run by using the isotype IgG antibody for each fluorochrome (Supplementary Figures S1–S4). Olympus inverted fluorescent microscope (Olympus BX51) was used to scan the images at 20× and 40× with a scale of 200 μm and 100 μm, respectively. The images were blindly reviewed by two independent observers and cross-evaluated by a board-certified pathologist. The immunofluorescence intensity for each image was quantified using Image J software, keeping the threshold consistent for each image, and then, mean intensity for each protein of interest was calculated. Mean fluorescence intensity (MFI) for CD86, CD206, IL-33, IL-37, TLR-2, and TLR-4 was quantified using three images from each swine in each group (15 images in VDDef group, 30 images in VDSuff group, and 15 images in VDSupp group) by Image-J software (NIH). The number of dual positive cells for CD14 with CD86 and CD206 were counted in three images in all swine and the average number of dual positive cells/mm^2^ was calculated for each group. The change in macrophage density in all three groups was also calculated relative to the average of M1 macrophage in VDDef group. For this, the average density of CD86 was calculated in VDDef swine and then the density of macrophages in each group was normalized to the average of M1 macrophage in VDDef group to calculate the relative change in macrophage density with different vitamin D status.

### 2.4. Stimulation and Inhibition Studies

The effect of calcitriol, IL-33, recombinant (r)HMGB-1, and LPS on the expression profile of IL-33, IL-37, HMGB-1, RAGE, TLR-2, TLR-4, IL-6, TNF-α, NF-κB, MMP-2, and MMP-9 was assessed by in vitro studies using normal human articular chondrocytes (NHAC). The effect of calcitriol on the effect of IL-33, rHMGB-1, and LPS on the mediators of inflammation was also investigated. NHAC cells were cultured in a T-75 flask and approximately 200,000 cells were plated in 6-well plates. After 80–90% confluence, cells were treated with calcitriol (50 nM), recombinant human IL-33 cytokine (25 ng/mL), recombinant human HMGB-1 (500 ng/mL), and LPS (100 ng/mL) for 24 h. From the control and treated cells, total RNA and cDNA were prepared, and qRT-PCR studies were conducted following standard protocols in our lab [42]. The forward and reversed primers used for qRT-PCR studies are shown in Table 1. The primers were designed using NCBI primer blast (https://www.ncbi.nlm.nih.gov/ last accessed on 5 April 2021). While designing primers, the following were considered: GC content > 50%, Tm = 60 ± 50 C, 20–22 base pair long, and amplicon length less than 250–300 base. The folds change in mRNA expression in the treatment group compared to the control for TLR-2, TLR-4, IL-6, TNF-α, NF-κB, HMGB-1, RAGE, MMP-2, and MMP-9 were analyzed after standardizing with GAPDH. All values are represented as mean ± SD. A value of *p* < 0.05 (* *p* < 0.05, ** *p* < 0.01, *** *p* < 0.001 and **** *p* < 0.0001) was considered statistically significant.

### 2.5. Cell Culture and Macrophage Polarization Studies

Human monocytes (THP-1 cells) were cultured and propagated in a T25 flask using the RPMI complete medium (5% fetal bovine serum + 1% penicillin-streptomycin) in a humidified incubator with 5% CO_2_ at 37 °C. At 80% confluence, 200,000 cells were plated in each well of a 6-well plate and treated with Phorbol 12-myristate 13-acetate (PMA) for 48 h to convert the monocytes (THP-1) to macrophages. After the cells attached themselves to the flask surface and transformed into macrophages, cells were treated with calcitriol (100 nM) for 6 days, changing the media every second day with a fresh treatment. After 6 days, the cells were trypsinized and subjected to flow cytometry to analyze the positively stained cells for CD86 for M1 macrophages and CD206, CD163, and IL-10 for M2a, M2b, and M2c macrophages, respectively, using the standard protocol. Briefly, the cells were washed with PBS4 (PBS + 4% fetal bovine serum) and centrifuged (300× *g* 10 min), followed by incubation with primary antibodies for anti-mouse CD86 PE-Cyanin5 (isotype IgG2bκ PE-Cyanin5), anti-rat CCR7 APCeFluor780 (isotype IgG2aκ APCeFluor780), anti-mouse CD206 Alexa Fluor 488 (isotype IgG1κ Alexa Fluor 488), anti-mouse CD163 Per-CP-eFluor 710 (isotype IgG1κ Per-CP-eFluor 710), and anti-mouse IL-10 PE-Cyanin 7 (isotype IgG1κ PE-Cyanin7) for 45 min at a concentration of 10 μL/106 cells. Cells were again centrifuged (300× *g* 10 min), and after removing the supernatant, 500 mL of FACS-fix (PBS:10% formaldehyde = 3:2) was added to the cells. Isotypes for each fluorochrome were used for negative control. OneComp eBeads (eBioscience 01-1111-42) with fluorescently conjugated antibodies were used for positive control. Live cells were gated using FSC/SSC. Cell populations were analyzed using Flow-Jo (v10) software. The average count of positively stained cells from the three separate experiments was calculated and analyzed by SPSS software for significance.

### 2.6. Statistical Analysis

Results are presented as mean ± SD (*n* = 3) for each parameter. One-way ANOVA (SPSS) with Bonferroni correction and a two-tailed student’s t-test was used for the significance of various parameters between two groups and among VDDef, VDSuff, and VDSupp. A *p*-value of <0.05 was considered significant. * *p* < 0.05, ** *p* < 0.01, *** *p* < 0.001 and **** *p* < 0.0001.

## 3. Results

### 3.1. Increased Immunopositivity for IL-33 and IL-37 in Vitamin D Deficient Microswine Cartilage

**Figure 1:**
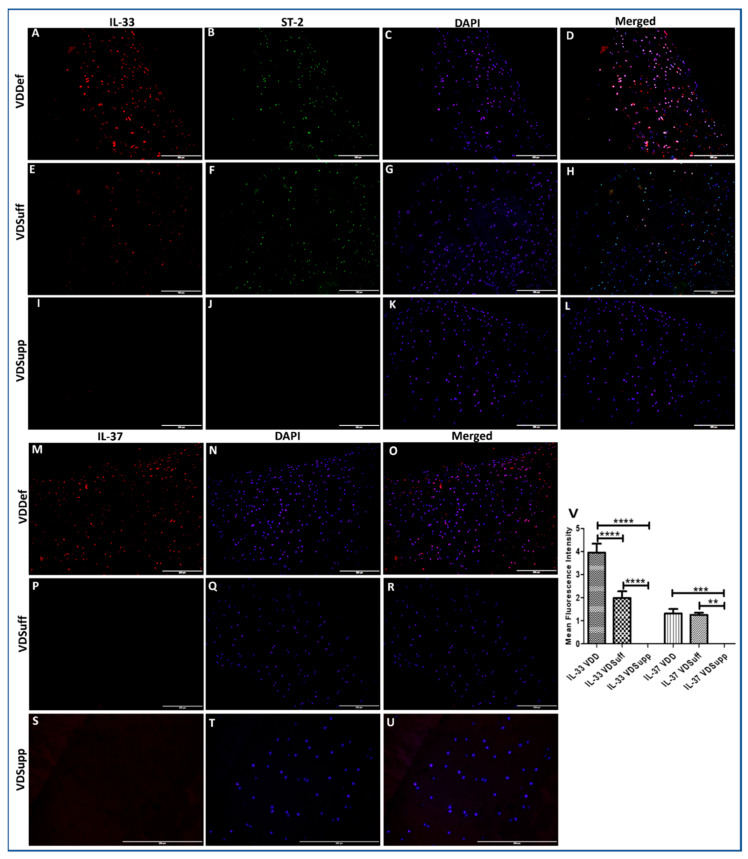
Immunofluorescence for IL-33 and IL-37 in VDDef, VDSuff, and VDSupp microswine cartilage. IL-33 (panels **A**,**E**,**I**), ST2 (panels **B**,**F**,**J**), IL-37 (panels **M**,**P**,**S**), DAPI (panels **C**,**G**,**K**,**N**,**Q**,**T**), merged images (panels **D**,**H**,**L**,**O**,**R**,**U**), and mean fluorescence intensity of IL-33 and IL-37 (panel **V**). These are the representative images from all subjects in the study. Results are presented as mean ± SD (*n* = 3). ** *p* < 0.01, *** *p* < 0.001 and **** *p* < 0.0001. DAPI—2–4-diamidinophenyl-1H-indole-6-carboxamidine, IL—interleukin, VDDef (vitamin D deficient), VDSuff (vitamin D sufficient), and VDSupp (vitamin D supplemented).

IF showed increased immunopositivity for IL-33 in VDDef compared to VDSuff and VDSupp and in VDSuff compared to VDSupp swine (Figure 1, panels A, E, and I). The immunopositivity for IL-37 in VDDef was higher compared to VDSuff and VDSupp swine (Figure 1, panels M, P, and S). IL-33 in VDSupp (Figure 1, panel I) and IL-37 in VDSuff (Figure 1, panel P) and VDSupp (Figure 1 panel S) showed no immunopositivity in the articular cartilage characterized by immunopositivity for chondrocyte-specific markers, namely collagen II, Sox-9, and chitinase-3 (Supplementary Figure S5). The mean fluorescence intensity for IL-33 and IL-37 was significantly higher in the VDDef group compared to VDSuff and VDsupp group and in the VDSuff group compared to the VDSupp group (Figure 1, panel V). These results indicate the association of the increased expression of IL-33 with vitamin D deficiency and decreased IL-33 and IL-37 expression with vitamin D supplementation (Figure 1).

### 3.2. Increased Immunopositivity for TLR-2 and TLR-4 in Vitamin D-Deficient Microswine Articular Cartilage

**Figure 2:**
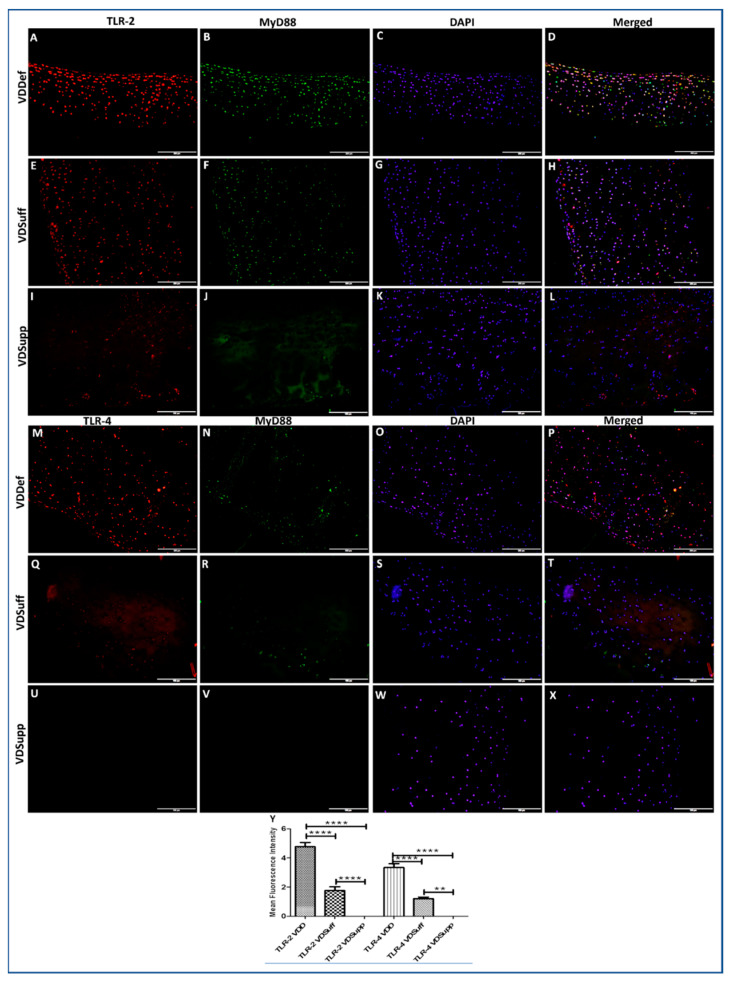
Immunofluorescence for TLR-2 and TLR-4 in VDDef, VDSuff, and VDSupp microswine cartilage. TLR-2 (panels **A**,**E**, and **I**), TLR-4 (panels **M**,**P**, and **S**), MyD88 (panels **B**,**F**,**J**,**N**,**R**, and **V**), DAPI (panels **C**,**G**,**K**,**O**,**S**, and **W**), merged images (panels **D**,**H**,**L**,**P**,**T**, and **X**), and mean fluorescence intensity of TLR-2 and TLR-4 (panel **Y**). These are the representative images from all the subjects in the study. Results are presented as mean ± SD (*n* = 3). ** *p* < 0.01, **** *p* < 0.0001. DAPI—2–4-diamidinophenyl-1H-indole-6-carboxamidine, MyD88—myeloid differentiation primary response gene 88, TLR—toll-like receptor.

The IF of VDDef, VDSuff, and VDSupp microswine articular cartilage showed higher immunopositivity for TLR-2 in VDDef compared to VDSuff and VDSupp (Figure 2, panels A, E, and I) and in VDSuff compared to VDSupp (Figure 2, panels E and I) microswine cartilage. Similarly, the immunopositivity for TLR-4 was higher in VDDef compared to VDSuff and VDSupp (Figure 2, panels M, P, and S). There was no immunopositivity for TLR-4 in VDSuff and VDSupp articular cartilage. The mean fluorescence intensity for TLR-2 and TLR-4 was significantly higher in the VDDef group compared to the VDSuff group and in the VDSuff group compared to the VDSupp group (Figure 2, panel Y). These results suggest the association of increased TLR2 and TLR4 with vitamin D deficiency and attenuation with vitamin D supplementation in the cartilage (Figure 2).

### 3.3. Increased Expression of M2 Macrophage in Vitamin D Deficient Microswine Cartilage

**Figure 3:**
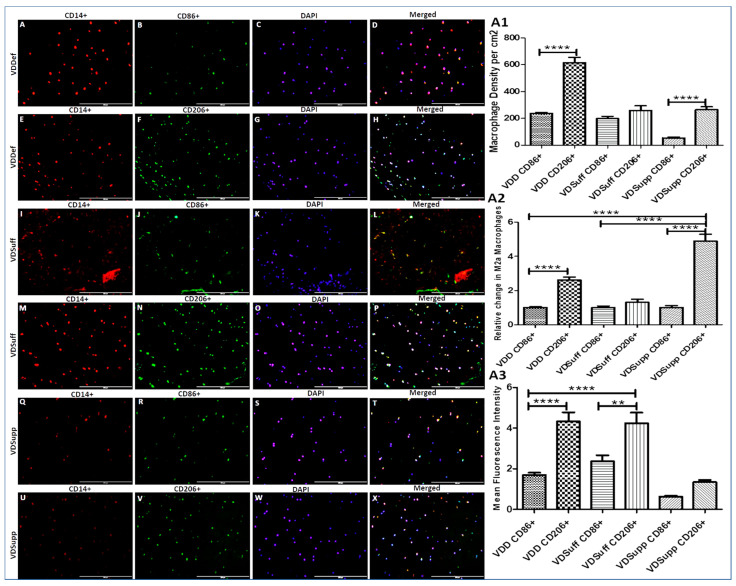
Immunofluorescence for M1 and M2 macrophages in VDDef, VDSuff, and VDSupp hyperlipidemic microswine. CD14+ cells (macrophages; panels **A**,**E**,**I**,**M**,**Q**, and **U**), CD86+ cells (M1 macrophages; panels **B**,**J**, and **R**), CD206+ cells (M2a macrophages; panels **F**,**N**, and **V**), DAPI (panels **C**,**G**,**K**,**O**,**S**, and **W**), merged images (panels **D**,**H**,**L**,**P**,**T**, and **X**), macrophage density (panel **A1**), the relative change in macrophage density with vitamin D status (panel **A2**), and mean fluorescence intensity of M1 and M2 macrophages (panel **A3**). These are the representative images of all the subjects in the study. Results are presented as mean ± SD (*n* = 3). ** *p* < 0.01, **** *p* < 0.0001. DAPI—2–4-di-amidinophenyl-1H-indole-6-carboxamidine.

Immunopositivity for CD14, CD86, CD206, and co-localization of CD14 with CD86 and CD206 in VDDef, VDSuff, and VDSupp microswine cartilage (Figure 3) suggests the presence of macrophages in the cartilage tissue. The results showed a higher density of macrophages in VDDef compared to VDSuff and VDSupp and in VDSuff compared to VDSupp microswine (Figure 3, panels A, E, I, M, Q, and O). Furthermore, there was a higher expression and density of M2 macrophages in VDDef (Figure 3, panel M) compared to VDSuff (Figure 3, panel P) and VDSupp (Figure 3, panel S) and in VDSuff (Figure 3, panel P) compared to VDSupp (Figure 3, panel S). IF also revealed that M1 macrophage expression was higher in VDDef compared to VDSuff and VDSupp and in VDSuff compared to VDSupp microswine. These results suggest the association of the increased expression of M1 and M2 density with vitamin D deficiency and a decrease with vitamin D supplementation (Figure 3, panels A1, A2, and A3). Since the articular cartilage is an avascular tissue, to elucidate the factors responsible for macrophage recruitment, we stained VDDef, VDSuff, and VDSupp microswine cartilage for CC chemokine receptor (CCR) 2, CC Motif Chemokine Ligand (CCL) 3, CCR5, CCR7, and monocytes chemoattractant protein (MCP)-1. Immunopositivity for these proteins (Supplementary Figure S6, panels A–O) suggests the presence of a chemokine receptor, chemokine ligands, and attractant proteins specific for monocyte and macrophage recruitment in the degenerating VDDef cartilage tissue.

### 3.4. Expression of VDR, Cyp24A1, and Cyp27B1 in Swine Cartilage

This study hypothesizes that vitamin D supplementation decreases inflammation and for that, vitamin D receptors should be present in the cartilage tissue. Therefore, we stained the VDDef, VDSuff, and VDSupp microswine cartilage for the vitamin D receptor (VDR) and the enzymes involved in vitamin D metabolism (cyp24A1 and Cyp27B1). The immunopositivity for VDR (Supplementary Figure S7, panel A), Cyp24A1 (Supplementary Figure S7, panel D), and Cyp27B1 (Supplementary Figure S7, panel G) suggests the presence of these proteins in the articular cartilage and indicates that vitamin D may be used therapeutically. To further validate the presence of these proteins in the chondrocytes, we stained normal human articular chondrocytes (NHAC) and human chondrocytes osteoarthritic (HCOA) cells for these proteins, and immunopositivity of these proteins in chondrocytes suggests the presence of these proteins in the chondrocytes (Supplementary Figure S8). The immunopositivity for VDR, Cyp24A1, and Cyp27B1 was higher in HCOA compared to NHAC cells and the immunopositivity for Cyp24A1 and Cyp27B1 was higher than VDR in NHAC and HCOA cells.

### 3.5. Calcitriol Attenuates the mRNA Expression of IL-33, TLR-2, TLR-4, NF-κB, IL-6, TNF-α, HMGB-1, RAGE, MMP-2, and MMP-9 in Normal Human Chondrocytes

**Figure 4:**
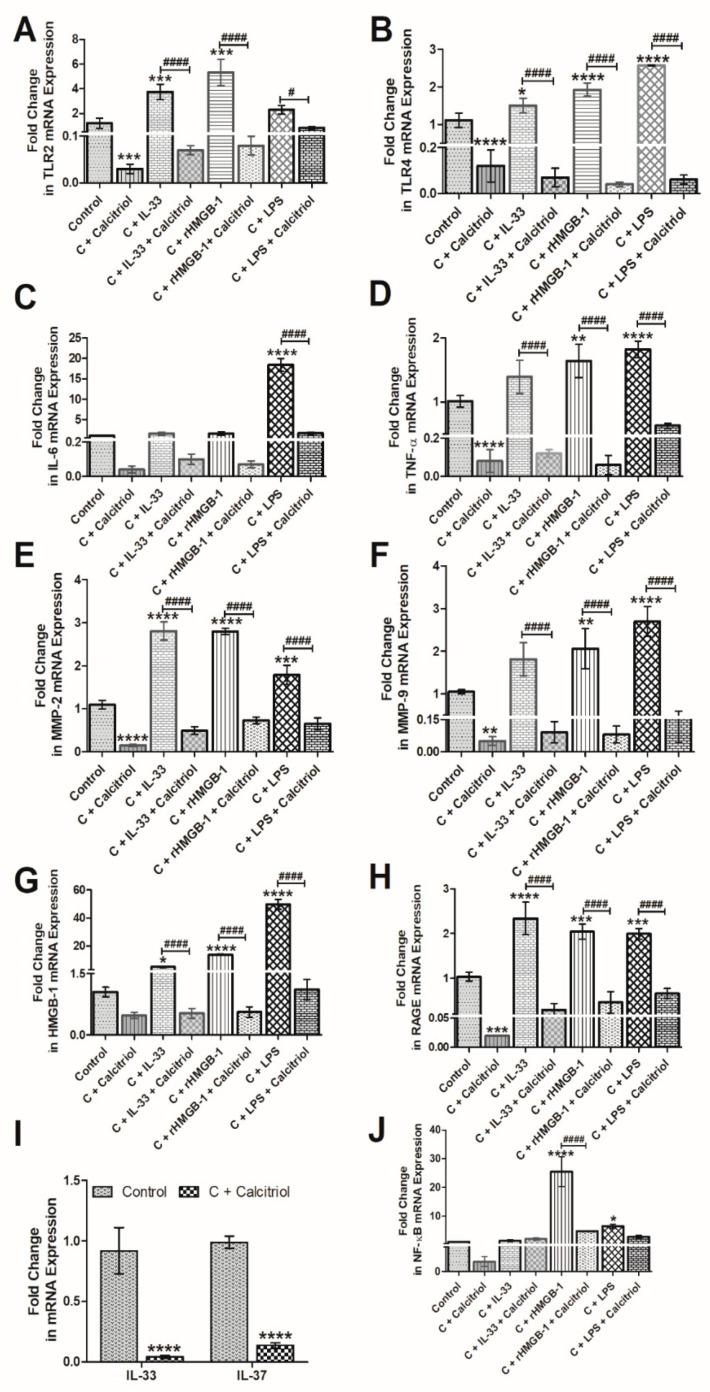
RT-qPCR analysis for the effect of calcitriol on the mRNA expression of mediators of inflammation. TLR-2 (panel **A**), TLR-4 (panel **B**), IL-6 (panel **C**), TNF-α (panel **D**), MMP-2 (panel **E**), MMP-9 (panel **F**), HMGB-1 (panel **G**), RAGE (panel **H**), IL-33 and IL-37 (panel **I**), and NF-κB (panel **J**). HMGB1- high mobility group box 1, IL-interleukin, MMP-matrix metalloproteinases, NF-κB-nuclear factor kappa beta, RAGE- receptor for advanced glycation end products, TLR- toll-like receptor, TNFα- tumor necrosis factor-alpha. Results are presented as mean ± SD (*n* = 3). * *p* < 0.05, ** *p* < 0.01, *** *p* < 0.001 and **** *p* < 0.0001. # *p* < 0.05, #### *p* < 0.0001, * compared to control and # compared to treatment with calcitriol in the respective group.

To elucidate the effect of calcitriol on various mediators of inflammation, we treated the NHAC cells with calcitriol and analyzed the mRNA expression of various genes. The RT-qPCR analysis of cDNA from untreated and calcitriol-treated NHAC showed that calcitriol significantly downregulates the mRNA expression of TLR-2 (Figure 4, panel A), TLR-4 (Figure 4, panel B), IL-6 (Figure 4, panel C), TNF-α (Figure 4, panel D), MMP-2 (Figure 4, panel E), MMP-9 (Figure 4, panel F), HMGB-1 (Figure 4, panel G), RAGE (Figure 4, panel H), IL-33, and IL-37 (Figure 4, panel I), and nuclear factor kappa beta (NF-κB) (Figure 4, panel J). These results suggest that calcitriol decreases the expression of mediators of inflammation and MMPs. A decreased expression of IL-37 with calcitriol suggests a possible feedback mechanism of decreasing IL-37 expression by reducing inflammation.

### 3.6. Calcitriol Downregulates the Effect of Recombinant(r) IL-33, rHMGB-1, and LPS on TLR-2, TLR-4, NF-κB, IL-6, TNF-α, MMP-2, MMP-9, HMGB-1, and RAGE in NHAC

The qRT-PCR analysis of cDNA synthesized from untreated and NHAC treated with calcitriol (50 nM), rIL-33 (25 ng/mL), rHMGB-1 (500 ng/mL), and LPS (100 ng/mL) revealed that calcitriol significantly downregulates the stimulatory effect of rIL-33, rHMGB-1, and LPS on the mRNA expressions of TLR-2, TLR-4, TNF-α, MMP-2, MMP-9, RAGE, and HMGB-1 (Figure 4). These findings suggest that calcitriol not only attenuates the mRNA expression of mediators of inflammation but also attenuates the effect of IL-33, HMGB-1, and LPS on the mediators of inflammation.

### 3.7. Calcitriol Favors M2 Macrophage Polarization

**Figure 5:**
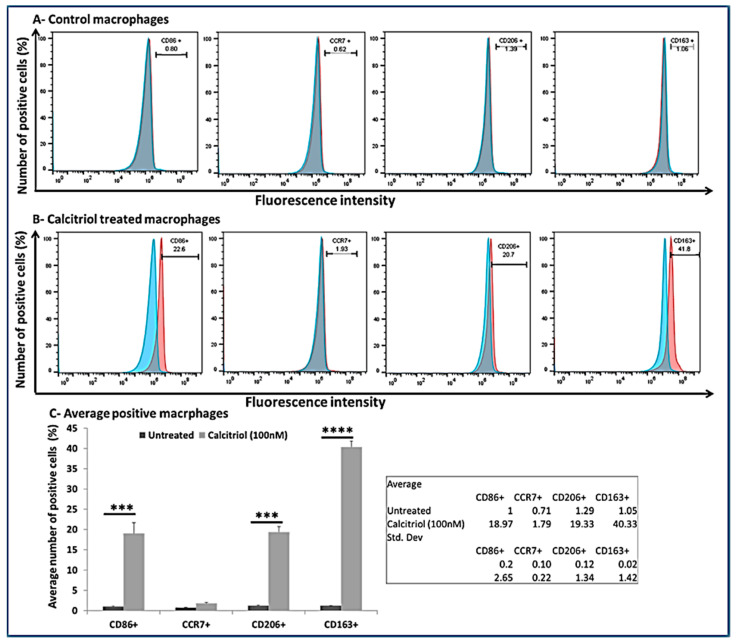
Flow cytometry analysis for the effect of calcitriol on PMA-treated THP-1 cells. (**A**) From left to right CD86+ cells, CCR7+ cells, CD206+ cells, andCD163+ cells in control macrophages, (**B**) From left to right CD86+ cells, CCR7+ cells, CD206+ cells, andCD163+ cells in calcitriol treated macrophages, (**C**) number of CD86, CCR7, CD206, CD163 positive cells (%). *** *p* < 0.001 and **** *p* < 0.0001. Blue color—isotype and red color—antigen-treated cells. CD—cluster differentiation, CCR—CC motif chemokine receptor.

To investigate the effect of calcitriol on macrophage polarization, we treated the macrophages with calcitriol (100 nM) and did the flow cytometry analysis. The results showed positivity for CD86, CCR7, CD206, and CD163 (Figure 5, panel A). The flow cytometry analysis revealed predominately M2 macrophages (CD206+ = 19.33 ± 1.34 %; CD163+ = 40.33 ± 1.42%) compared to M1 macrophages (CD86+ = 18.97 ± 2.65%; CCR7+ = 1.79 ± 0.22%) suggesting a M2 macrophage polarization with calcitriol (Figure 5, panels B and C).

## 4. Discussion

The results of this study showed immunopositivity for VDR, Cyp24A1, Cyp27B1, collagen II, sox-9, and chitinase-3 in swine cartilage. These results characterize the nature of the cells present in the tissue as chondrocytes and the tissue as cartilage [43] and indicate the presence of vitamin D receptors and enzymes involved in the metabolism of vitamin D in the cartilage. This suggests that vitamin D may act as an immunomodulator in the cartilage tissue [44,45]. The IF studies also revealed significantly increased immunopositivity for IL-33, IL-37, TLR-2, TLR-4, M1 macrophage (CD86), and M2 macrophage (CD206) in VDDef swine cartilage compared to VDSuff and VDSupp microswine cartilage. These results suggest the presence of increased inflammation in the degenerating cartilage of VDDef swine [1]. In our previous study, the increased expression of MMPs decreased the expression of collagens, and degenerating histology of cartilage in VDDef swine was documented [1]. The increased expression of IL-37 and M2 macrophages (CD14+CD206+ cells) suggests the innate immune response of the body against the ongoing inflammation in the cartilage [10,46,47,48]. The increased expression of the IL-33, TLRs, and M1 macrophages in VDDef swine suggests the presence of inflammation and increased cartilage loss associated with vitamin D deficiency [1]. Further, the association between vitamin D deficiency and the higher prevalence of OA in elderly patients and poor outcomes after total knee replacement surgery [49] supports these results and the notion that vitamin D supplementation might be beneficial in OA for better clinical outcomes.

The presence of macrophages within the degenerating cartilage in association with mediators of inflammation and the role of synovial macrophages in the pathogenesis of synovitis and OA has been documented [1,50]. Pro-inflammatory macrophages (M1 or classically activated macrophages) play a central role in the host defense against infection and anti-inflammatory macrophages (M2 or alternatively activated macrophages) play a central role in anti-inflammatory response and tissue remodeling [10,48]. The density of M2 macrophages predominates in the later phase of inflammation to subside the inflammatory response. In this study, IF revealed the higher expression of M1 macrophages (CD86+ cells) and M2 macrophages (CD206+ cells) in VDDef swine compared to VDSuff and VDSupp swine and in VDSuff swine compared to VDSupp swine. A significant decrease in the expression of M1 macrophages (CD86+ cell) in VDSuff swine and no expression of M1 and M2 macrophages in VDSupp swine suggest the immunomodulatory role of vitamin D and its role in macrophages polarization. M2 macrophages are anti-inflammatory macrophages and are predominant in the late phase of inflammation. Vitamin D is also an anti-inflammatory agent and favors M2 macrophage predominance [51,52]. These results suggest the attenuation of inflammation and macrophage phenotypic switching with vitamin D supplementation. The presence of the M2 macrophage in VDDef swine may be due to the innate defense mechanism of the body to fight against inflammation, while in VDSuff swine, it may be due to the immunomodulatory effect of vitamin D given to the swine [44].

The process of initiation, development, and cessation of inflammation is regulated by the transformation of macrophages into different phenotypes, termed macrophage polarization [10,48]. Monocyte to macrophage differentiation and phenotypic switching of macrophages is regulated by various factors, including lipopolysaccharides (LPS), interferon (IFN)-γ, IL-4, IL-13, IL-1β, IL-10, vitamin D, and transforming growth factor (TGF)-β [10,11,53]. Furthermore, the polarization towards classically and alternatively activated macrophages by IL-33 and towards the alternatively activated macrophages by IL-37 has been discussed [12,13,54,55,56,57]. These studies suggest the role of IL-33, IL-37, and vitamin D in macrophage differentiation and polarization. The presence of IL-33 and macrophages in vitamin D-deficient degenerating cartilage of microswine and the presence of macrophages in human osteoarthritic cartilage suggests a correlation between IL-33 and vitamin D deficiency in OA. An association of vitamin D deficiency with the incidence and prevalence of OA suggests that the interaction of these molecules may play a pathogenic and therapeutic role in OA [21,24,33,46,58,59,60]. Vitamin D is an inducer of M2 macrophages and has a VDR-dependent response. Vitamin D deficiency and VDR deletion are associated with chronic inflammation, partially due to altered M1/M2 polarization [52,61]. In our study, flow cytometry data of the calcitriol-treated macrophages showed a predominance of M2 macrophages (CD206+ = 19.33 ± 1.34% and CD163+ = 40.33 ± 1.42%) compared to M1 macrophages (CD86+ = 18.97 ± 2.65% and CCR7+ = 1.79 ± 0.22%). These results suggest that calcitriol favors M2 phenotype polarization. Since M2 macrophages are considered to have an anti-inflammatory function, increased M2 macrophages in VDSuff, no expression of M2 macrophages in VDSupp, and increased M2 with calcitriol suggest the anti-inflammatory role of vitamin D via targeting the M1/M2 polarization towards M2 macrophages. These results agree with previous studies reporting decreased inflammation with vitamin D supplementation [1,48,57].

The amelioration of the clinical symptoms of experimental autoimmune encephalomyelitis along with decreased IL-33 expression with vitamin D suggests the immunomodulatory and anti-inflammatory effects of vitamin D [15]. Vitamin D deficiency is associated with the loss of bone mineralization and decreased bone mineral density (BMD) which, in turn, is associated with an increased incidence of OA [62]. Since vitamin D supplementation increases bone mineralization and bone mineral density (BMD) [63], the supplementation of vitamin D may decrease cartilage loss; however, the loss of cartilage volume may be dose-dependent. Higher levels of vitamin D may not prevent the progression of knee pain and cartilage volume loss and the development of OA of the knee joint [35,64]. It has been reported that vitamin D, through a post-transcriptional mechanism, has a dose-dependent regulation of chondrocyte gene expression, and higher levels may result in a more rapid turnover of the aggrecan mRNA, resulting in a decreased synthesis of aggrecan [65].

**Figure 6:**
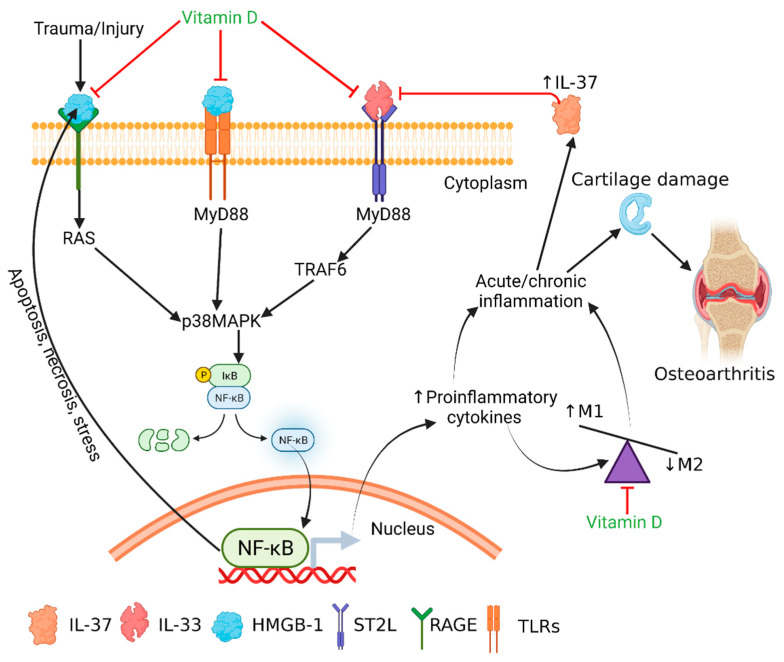
Schematics predicting the IL-33-mediated inflammation and the therapeutic targets of calcitriol. HMGB-1 is secreted after the initial damage to the cartilage, which also increases secretion of IL-33. Downstream signaling of HMGB-1 through RAGE and TLRs and of IL-33 through NF-kB leads to the increased secretion of pro-inflammatory cytokines, resulting in acute inflammation. Persistent secretion of the inflammatory cytokines such as IL-33 may result in chronic inflammation in the cartilage and continued cartilage damage, leading to severe OA. Vitamin D attenuates the downstream signaling of HMGB-1 and IL-33 and suppresses the secretion of inflammatory cytokines. Vitamin D also favors M2 macrophage polarization, having an anti-inflammatory action. Thus, vitamin D might be used to target IL-33- and HMGB-1-mediated inflammation in osteoarthritis.

Increased expression of IL-33, TLR-2, and TLR-4 in VDDef swine suggests an increased level of inflammation within the cartilage. HMGB-1, IL-33, and TLRs-mediated downstream signaling enhances the secretion of pro-inflammatory cytokines, including IL-4, IL-5, IL-6, IL-8, and IL-13 and mediates chronic inflammation [18,19]. Increased IL-6 secretion from macrophages and its detrimental role in OA has been documented in the literature [66]. The attenuation of IL-33 expression in VDSuff and VDSupp swine suggests the anti-inflammatory role of vitamin D and the possible role of vitamin D in attenuating inflammation in OA (Figure 1 and Figure 6). The downregulation of mRNA expression of IL-33 in calcitriol-treated NHAC cells further supports the immunomodulatory role of vitamin D (Figure 4). Vitamin D regulates the secretion as well as the inflammatory action of IL-33 and attenuates its expression and action by inducing the secretion of soluble ST2, a decoy receptor of IL-33 [16,67]. Vitamin D3 inhibits the IL-33 expression in IL-4 stimulated epithelial cells and decreases the IL-33 expression in LPS-stimulated cells [16]. In this study, calcitriol downregulated the IL-33 mRNA expression in IL-33, rHMGB-1, and LPS-stimulated cells as well as the stimulatory effect of IL-33, rHMGB-1, and LPS on TLR-2, TLR-4, IL-6, TNF-α, NF-κB, HMGB-1, RAGE, MMP-2, and MMP-9 (Figure 4). These results suggest the inhibitory effect of calcitriol on the expression and action of IL-33. Further, no immunopositivity for IL-33, TL-2, and TLR-4 in VDSupp swine suggests that supplementing vitamin D may be beneficial in decreasing chronic inflammation and thereby cartilage damage [1,14]. These findings are further supported by the decreased cartilage loss and macrophage density in VDSupp compared to VDSuff and VDDef and in VDSuff compared to VDDef swine reported in our previous study [1].

IL-37 is an anti-inflammatory modulator in obesity and attenuates inflammation via the activation of AMPK signaling, decreased secretion of pro-inflammatory cytokines, decreased recruitment of pro-inflammatory cells, and attenuating the downstream pro-inflammatory signaling through TLRs in mice [46]. IL-37 has an anti-inflammatory effect in rheumatoid arthritis [7,66,68,69], reduces the secretion of IL-1β, IL-6, and TNF-α and MIP-2, and mediates M2 macrophage polarization [57,70]. Further, enhancement of the immunological defense via the increased expression of VDR and IL-37 in peritoneal macrophages with vitamin D supplementation suggests that the vitamin D-VDR-IL-37 axis in macrophage plays a key role in innate immunity [71]. Immunofluorescence studies of the swine cartilage in this study revealed a higher expression of IL-37 in VDDef swine compared to VDSuff and VDSupp swine. There was no immunopositivity for IL-37 in VDSuff and VDSupp swine. Furthermore, the qRT-PCR analysis showed that calcitriol attenuated the expression of IL-37 in NHAC cells. These results suggest that the secretion of IL-37 in response to ongoing inflammation as a defense mechanism of the body decreases with the subsiding inflammation with vitamin D [72,73].

Vitamin D is an anti-inflammatory and immunomodulatory agent; supplementation of vitamin D associated with no expression of IL-37 in the cartilage of VDSuff and VDSupp swine can be explained by the fact that vitamin D supplementation decreases inflammation and results in an attenuated secretion of IL-37. However, studies have also reported that vitamin D may induce the expression of IL-37 and IL-37 may act as a mediator for the action of vitamin D [47,72]. The nuclear translocation of IL-37, its release in inflammasome, and anti-inflammatory activity depend on caspase-1 activation. The secretion of pro-inflammatory cytokines, including IL-6 and IL-1β and chemokines from macrophages, is attenuated by IL-37 in a nucleotide-binding oligomerization domain-like receptor family, pyrin domain containing 3 (NLRP3)-dependent manner [74]. However, Tulk et al. reported the enhanced secretion of IL-1β in an NLRP3-dependent manner under the influence of vitamin D from THP-1 cells [75]. Thus, an in-depth understanding of the IL-37-mediated attenuation of pro-inflammatory cytokines and the role of the vitamin D-VDR-IL-37 axis in chondrocytes is important for the development of novel therapeutic strategies.

The crucial role of IL-33 and IL-37 in the pathogenesis of OA has been elucidated in human osteoarthritic cartilage, and the results suggest that IL-33 mediates its pro-inflammatory action through TLR-2 and TLR-4, whereas IL-37 has an inhibitory effect on these TLRs [14]. The role of various TLRs has been discussed in the literature [76,77,78]; however, the effect of vitamin D on the TLRs in OA has not been discussed. In this study, IF revealed an increased expression of TLR-2 and TLR-4 in VDDef swine compared to VDSuff and VDSupp swine. The TLR-2 expression in VDSuff swine was higher than VDSupp swine but there was no immunopositivity for TLR-4 in VDSuff and VDSupp swine (Figure 2). The RT-qPCR analysis of the calcitriol-treated NHAC cells showed the attenuation of mRNA expressions of TLR-2 and TLR-4 (Figure 4). These results suggest that the expression of TLR-2 and TLR-4 decreases with vitamin D supplementation. The qRT-PCR analysis also revealed that calcitriol diminished the stimulatory effect of IL-33, rHMGB-1, and LPS on TLR-2 and TLR-4. The attenuation of TLRs with calcitriol and vitamin D supplementation suggests the anti-inflammatory role of vitamin D in suppressing not only TLRs but also pro-damage mediators MMPs and the action of IL-33, rHMGB-1, and LPS on these mediators [30,67,79,80,81,82,83,84]. Since DAMPs play a crucial role in the pathogenesis of OA [2,3], targeting these with vitamin D might be beneficial in attenuating inflammation and cartilage damage. Targeting IL-33 and increasing IL-37 in OA to attenuate inflammation and cartilage loss is supported by the fact that the cartilage-specific deletion of IL-33 improves disease outcomes and IL-33 signaling inhibition downregulates the release of cartilage-degrading enzymes. Furthermore, decreased proteoglycan loss in human OA cartilage with IL-37 via inhibition of MMP-3 expression and the protection of stem cells in an inflammatory osteoarthritis-like microenvironment for cartilage formation support IL-33 and IL-37 as potential therapeutics [20,85,86,87]. IL-33 and IL-37 play a counteractive role in regulating inflammation involving the activation of mast cells in chronic rhinosinusitis with a differential expression in allergy and sinusitis compared to polyp, which further suggests IL-33 and IL-37 as novel therapeutic targets, and the findings of this study support this [88]. The therapeutic efficacy of IL-37 in attenuating inflammation by inhibiting the transcription of pro-inflammatory genes by blocking IL-1 receptors [89]. The results of these studies support targeting IL-33 to attenuate inflammation and our study showing the decreased expression of IL-33 with IL-37 and vitamin D suggests vitamin D and IL-37 as potential therapeutics.

## 5. Conclusions

The results of this study suggest the presence of mediators of inflammation including HMGB-1, RAGE, IL-6, TNF-α, TLR-2, TLR-4, NF-κB, and MMPs in vitamin D-deficient swine cartilage and the attenuation of these proteins with vitamin D supplementation. Additionally, in vitro studies suggest the downregulation of mediators of inflammation with calcitriol. The results also suggested predominant M1 macrophages with vitamin D deficiency and favored M2 macrophages polarization with calcitriol. Taken together, these results are indicative of the immunomodulatory role of vitamin D in attenuating chronic inflammation by targeting IL-33-mediated inflammation in OA. Thus, vitamin D supplementation in patients with a vitamin D deficiency may be beneficial in treating patients with osteoarthritis (Figure 6) and may be a novel therapeutic strategy by blocking the effect of IL-33. However, for an in-depth understanding of molecular aspects of the IL33–IL37–vitamin D axis, endogenous factors regulating this axis and the dosages of vitamin D warrant future studies.

## 6. Limitations of the Study

This study provides significant results that vitamin D supplementation attenuates inflammation in degenerating cartilage and vitamin D supplementation might be used as an adjunct therapy in such patients. The non-availability of control swine cartilage is a major limitation of this study and further studies could be conducted including control swine on a normal diet. Despite this limitation, this study elucidated the pathogenic role of IL-33, TLR-2, and TLR-4 in OA and the beneficial role of calcitriol in OA.

## Figures and Tables

**Table 1:** Forward and reverse primer gene sequence used in qRT-PCR. Glyceraldehyde 3-phosphate dehydrogenase (GAPDH), high mobility group box (HMGB-1), interleukin (IL), matrix metalloproteinases (MMP), nuclear factor-kappa beta (NF-κB), the receptor for advanced glycation end products (RAGE), and tumor necrosis factor-alpha(TNF-α).

Gene of Interest	Forward Primer	Reverse Primer
HMGB-1	5′-AAG CAC CCA GAT GCT TCA GT-3′	5′-TCC GCT TTT GCC ATA TCT TC-3′
IL-33	5′-GTGACGGTGTTGATGGTAAGA -3′	5′-CTCCACAGAGTGTTCCTTGTT -3′
IL-37	5′-GCACCTCCTGCAATTGTAATG -3′	5′-CAGTTTCCTAATCGCTGACCT -3′
IL-6	5′-ATA GGA CTG GAG ATG TCT GAG G-3′	5′-GCT TGT GGA GAA GGA GTT CAT AG-3′
MMP-2	5′-TGA TGG TGT CTG CTG GAA AG-3′	5′-CTA CAG GAC AGA GGG ACT AGA G-3′
MMP-9	5′-ACA AGC TCT TCG GCT TCT G -3′	5′-GGT ACA GGT CGA GTA CTC CTT A-3′
NF-κB	5′-GAC TAC GAC CTG AAT GCT GTG-3′	5′-GTC AAA GAT GGG ATG AGG AAG G-3′
RAGE	5′- CCT GCA GGG ACT CTT AGC TG-3′	5′- CTC CGA CTG CAG TGT GAA GA-3′
TLR-2	5′-CTG GAG AAA GCC TTG AAC TCT AT-3′	5′-GAC ACT CGG TCT CTA GCA ATT T-3′
TLR-4	5′-TCA AAG AGC TGG TGC GAA A-3′	5′-CAG CTG CTT GTC TGC ATT TG-3′
TNF-α	5′-ACC CTC AAC CTC TTC TGG CTC AA-3′	5′-AAT CCC AGG TTT CGA AGT GGT GGT-3′
GAPDH	5′-GGT GAA GGT CGG AGT CAA CGG ATT TGG TCG-3′	5′-GGA TCT CGC TCC TGG AAG ATG GTG ATG GG-3′

## Data Availability

All data have been included in the manuscript.

## Supplementary Materials

The following are available online at https://www.mdpi.com/article/10.3390/antib10040041/s1, Figure S1: Positive controls in liver from vitamin D deficient microswine. Panel (A) TLR-2, panel (D) TLR-4, panel (G) IL-33, panel (K) IL-37, panel (N) CD14, panel (Q) CD86, panel (T) CD206, panels (B), (E), (I), (L), (O), (R), and (U) are DAPI, and panels (C), (F), (J), (M), (P), (S) and (V) are merged images. Figure S2: Positive controls in spleen from vitamin D deficient microswine. Panel (A) TLR-2, panel (D) TLR-4, panel (G) IL-33, panel (K) IL-37, panel (N) CD14, panel (Q) CD86, panel (T) CD206, panels (B), (E), (I), (L), (O), (R), and (U) are DAPI, and panels (C), (F), (J), (M), (P), (S) and (V) are merged images, Figure S3: Negative control using IgG isotype controls in cartilage tissue. Panel (A) anti-rabbit IgG, panel (D) anti-mouse-IgG, panel (G) anti-goat IgG, panels (B), (E), and (H) are DAPI, and panels (C), (F), and (I) are merged images. Figure 4: Negative control using only primary and only secondary anti-body. Panel (A) TLR-2 primary antibody alone; no secondary, panel (D) CD-14 primary antibody alone; no secondary, panel (G) secondary antibody Alexa-Fluor 594 only; no primary, panels (B), (E), and (H) are DAPI, and panels (C), (F), and (I) are merged images, Figure S5: Immunofluo-rescence for collagen II, Sox-9 and chitinase-3 in VDDef, VDSuff, and VDSupp hyperlipidemic microswine articular cartilage. Collagen II- panel (A), sox 9—panel (D), chitinase 3—panel (G), DAPI—panels (B), (E), and (H), merged images- panels (C), (F), and (I). DAPI—2-4-amidinophenyl-1H-indole-6-carboxamidine. These are the representative images from all the subjects in the study, Figure S6: Immunofluorescence for CCR2, CCL3, CCL5, CCR7, and MCP-1 in VDDef, VDSuff, and VDSupp microswine cartilage. CCL2—panel (A), CCL3—panel (D, CCR5—panel (G), CCR7—panel (J), MCP1—panel (M); DAPI—panels (B), (E), (H), (K), and (N); merged images (C), (F), (I), (L), and (O). These are the representative images of all the subjects in the study. CCL—C-C motif chemokine ligand, CCR—C-C motif chemokine receptor, DAPI—2-4-amidinophenyl-1H-indole-6-carboxamidine, MCP1—monocyte chemoattractant pro-tein 1, Figure S7: Immunofluorescence for VDR, Cyp24A1 and Cyp27B1 in VDDef, VDSuff, and VDSupp hyperlipidemic microswine articular cartilage. VDR- panel (A), Cyp24A1—panel (D), Cyp27B1—panel (G), DAPI—panels (B), (E), and (H), merged images—panels (C), (F), and (I). DAPI—2-4-amidinophenyl-1H-indole-6-carboxamidine, VDR—vitamin D receptor. These are the representative images from all the subjects in the study, Figure S8: Immunofluorescence for VDR, Cyp24A1 and Cyp27B1 in NHAC and HCOA cells. VDR—panels (A) and (D), Cyp24A1—panels (G) and (J), Cyp27B1—panels (M) and (P), DAPI—panels (B), (E), (H), (K), (N), and (Q), merged im-ages—panels (C), (F), (I), (L), (O), and (R). DAPI—2-4-amidinophenyl-1H-indole-6-carboxamidine, VDR—vitamin D receptor.
