# Supplementary material for: IL-33, IL-37, and Vitamin D Interaction Mediate Immunomodulation of Inflammation in Degenerating Cartilage
Source: Antibodies (Basel). 2021 Oct 26;10(4):41. doi: 10.3390/antib10040041 (PMC8628513; doi:10.3390/antib10040041)
Supplement: Supplementary file 1 [file antibodies-10-00041-s001.zip › antibodies-1376559-supplementary.pdf]

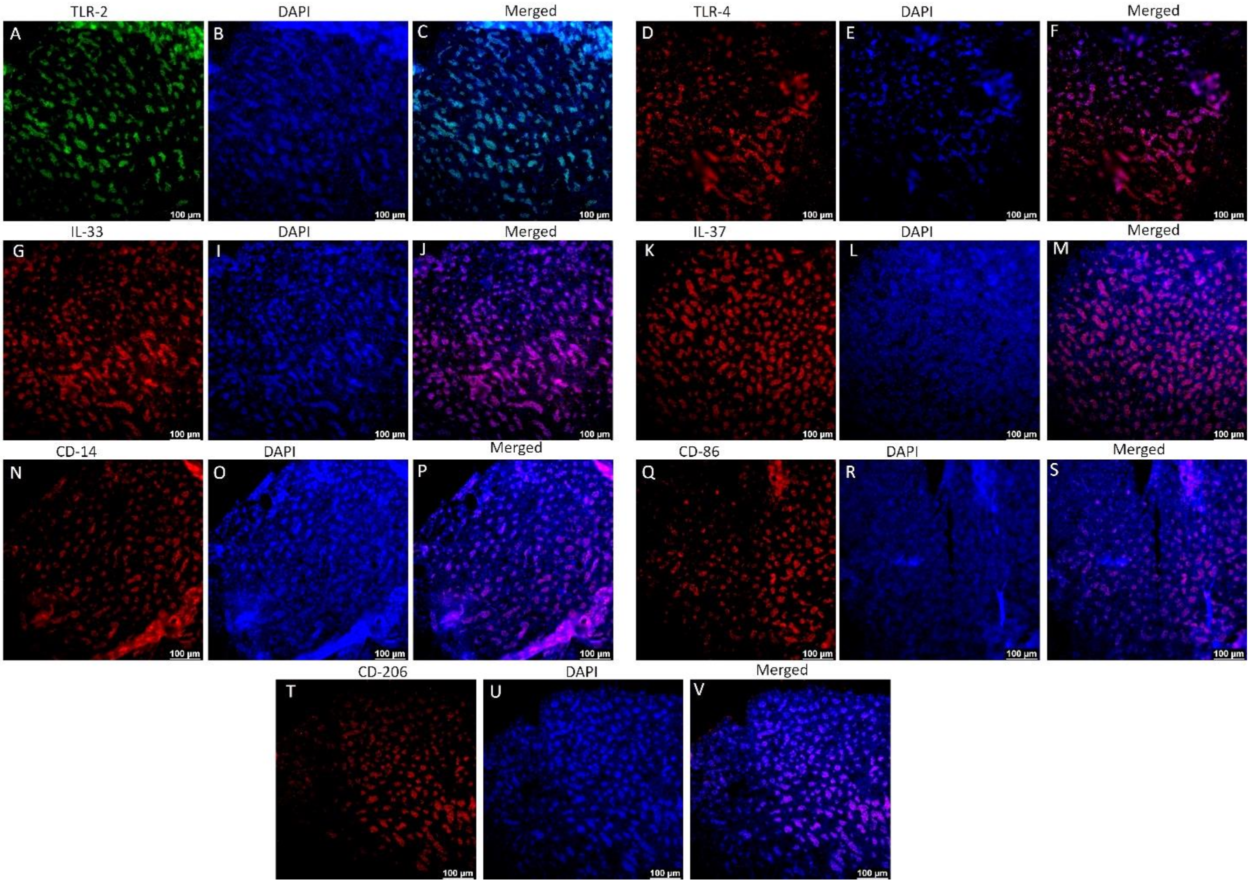

Figure S1. Positive controls in liver from vitamin D deficient microswine. Panel (A) TLR-2, panel (D) TLR-4, panel (G) IL-33, panel (K) IL-37, panel (N) CD14, panel (Q) CD86, panel (T) CD206, panels B, E, I, L, O, R, and U are DAPI, and panels C, F, J, M, P, S and V are merged images.

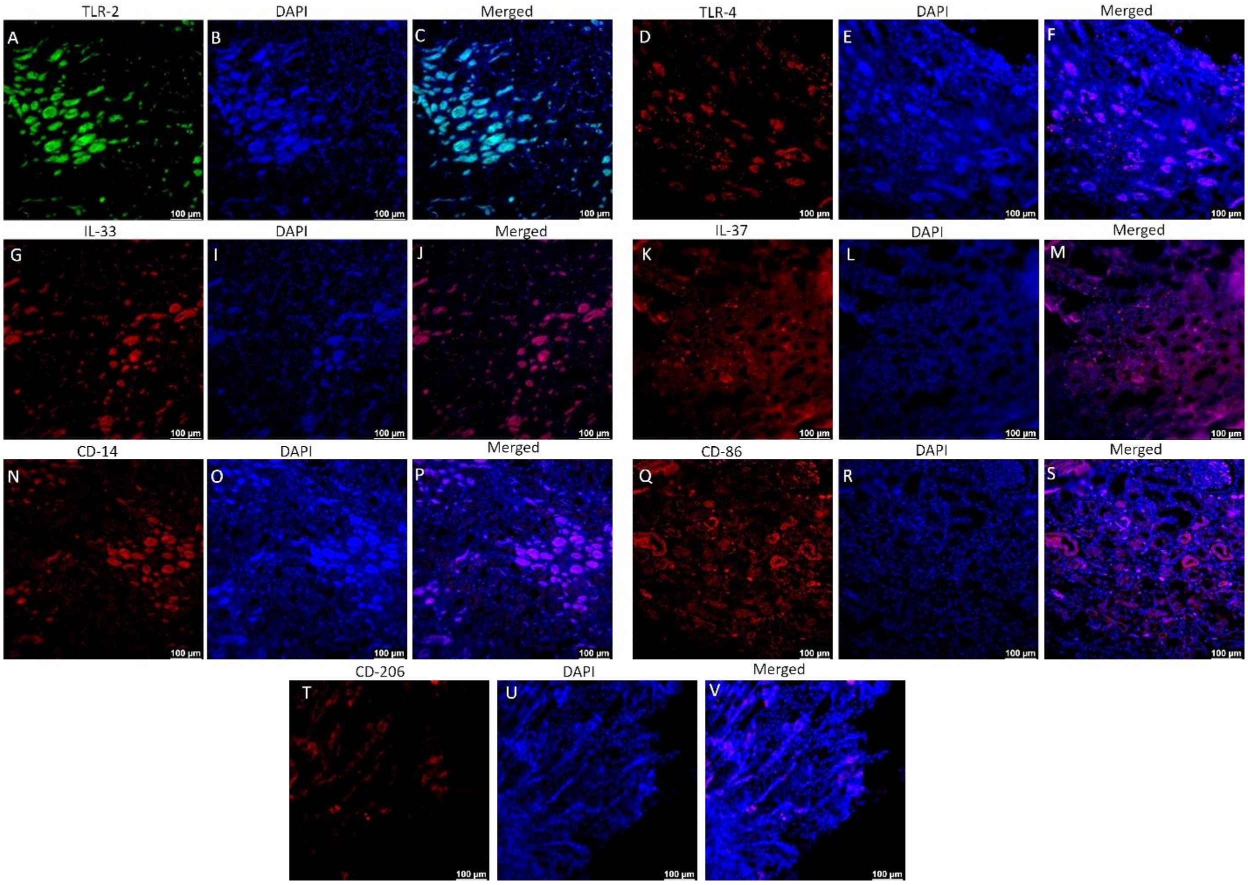

Figure S2. Positive controls in spleen from vitamin D deficient microswine. Panel A) TLR-2, panel D) TLR-4, panel G) IL-33, panel K) IL-37, panel N) CD14, panel Q) CD86, panel T) CD206, panels B, E, I, L, O, R, and U are DAPI, and panels C, F, J, M, P, S and V are merged images.

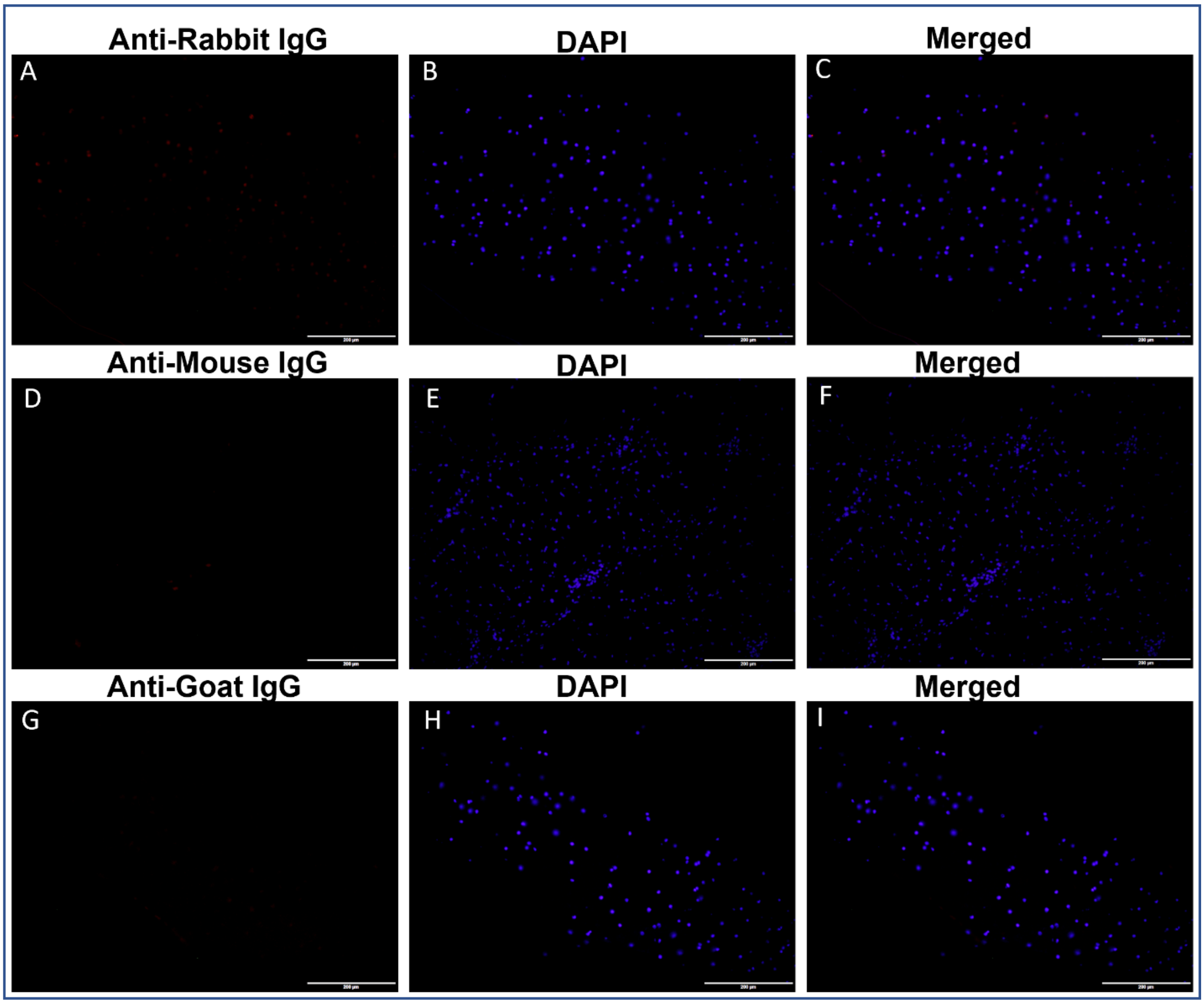

Figure S3. Negative control using IgG isotype controls in cartilage tissue. Panel A) anti-rabbit IgG, panel D) anti-mouse-IgG, panel G) anti-goat IgG, panels B, E, and H are DAPI, and panels C, F, and I are merged images.

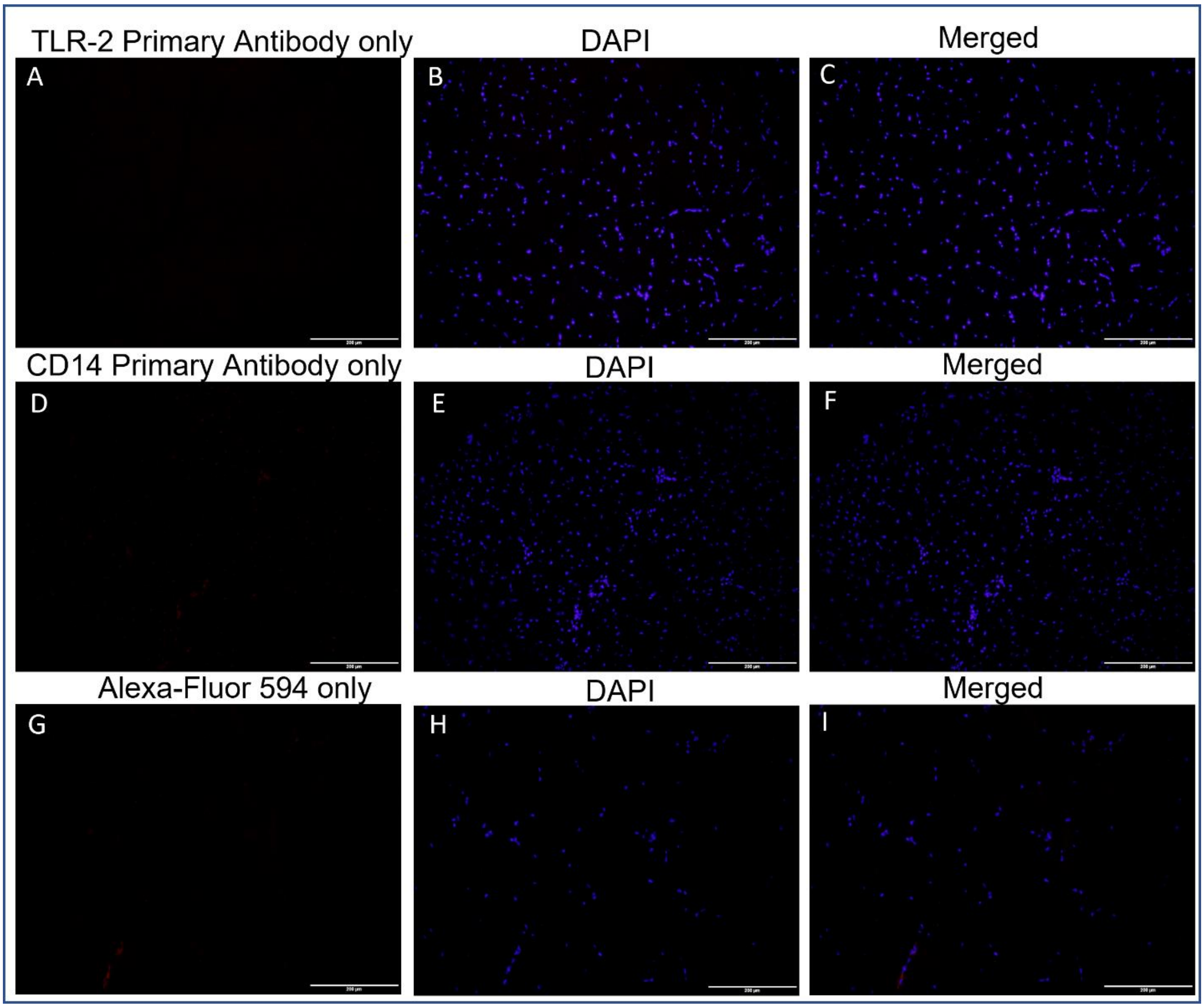

Figure S4. Negative control using only primary and only secondary antibody. Panel A) TLR-2 primary antibody alone; no secondary, panel D) CD-14 primary antibody alone; no secondary, panel G) secondary antibody Alexa-Fluor 594 only; no primary, panels B, E, and H are DAPI, and panels C, F, and I are merged images.

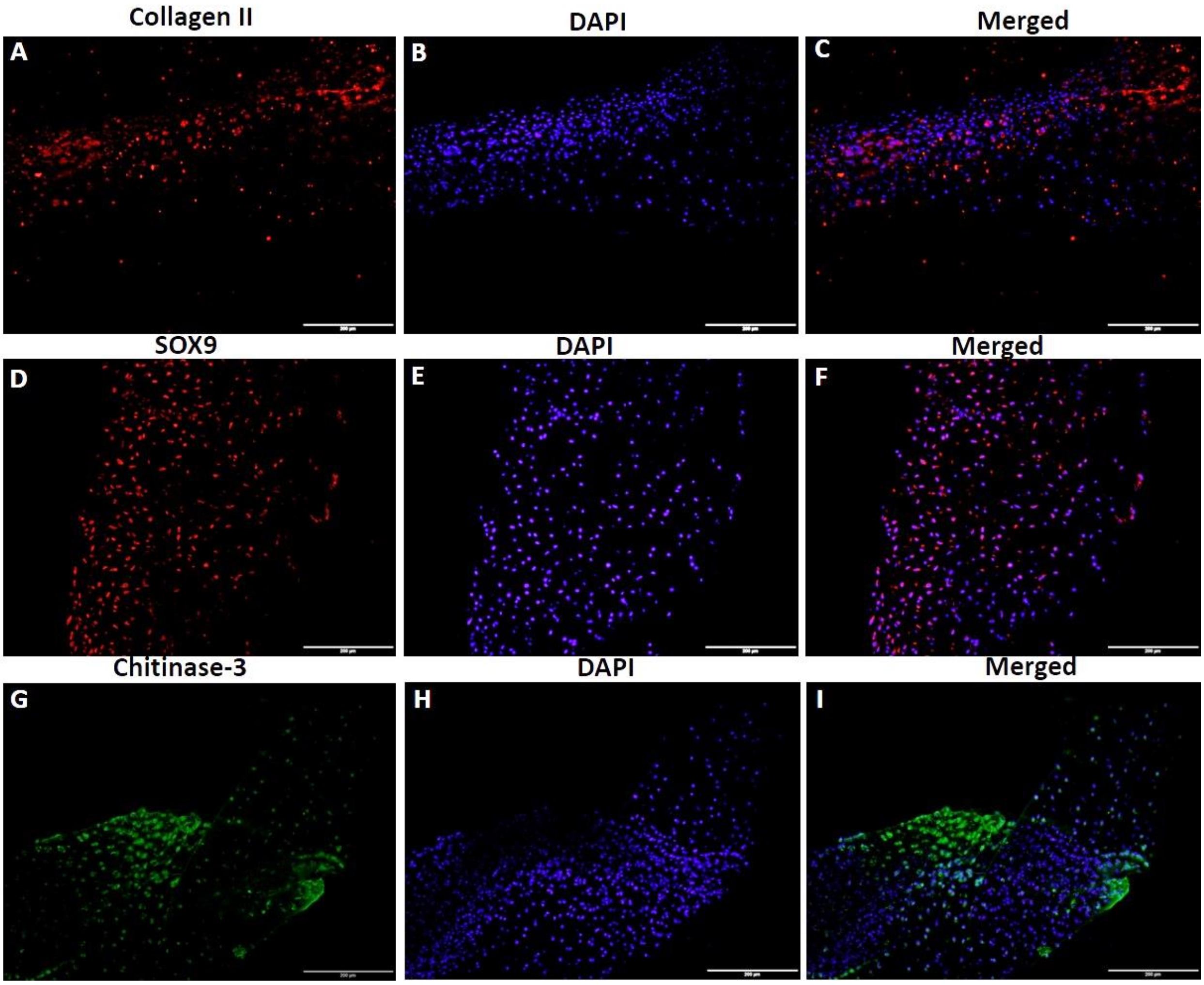

Figure S5. Immunofluorescence for collagen II, Sox-9 and chitinase-3 in VDDef, VDSuff, and VDSupp hyperlipidemic microswine articular cartilage. Collagen II- panel A, sox 9- panel D, chitinase 3- panel G, DAPI- panels B, E, and H, merged images- panels C, F, and I. DAPI- 2-4-amidinophenyl-1H -indole-6-carboxamidine. These are the representative images from all the subjects in the study.

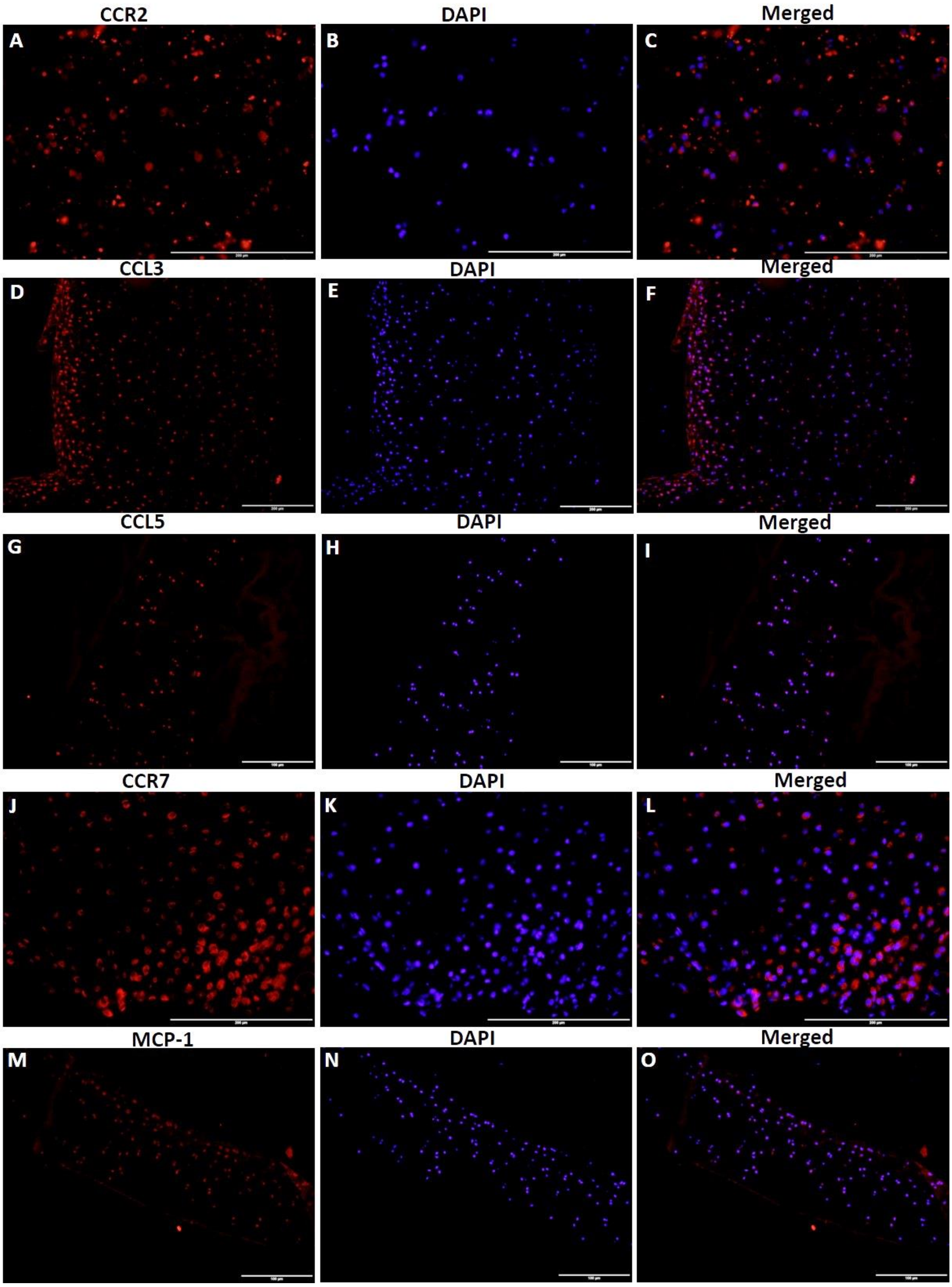

Figure 6. Immunofluorescence for CCR2, CCL3, CCL5, CCR7, and MCP-1 in VDDef, VDSuff, and VDSupp microswine cartilage. CCL2- panel A, CCL3- panel D, CCR5- panel G, CCR7- panel J, MCP1- panel M; DAPI- panels B, E, H, K, and N; merged images C, F, I, L, and O. These are the representative images of all the subjects in the study. CCL- C-C motif chemokine ligand, CCR- C-C motif chemokine receptor, DAPI- 2-4-amidinophenyl-1H -indole-6-carboxamidine, MCP1- monocyte chemoattractant protein 1.

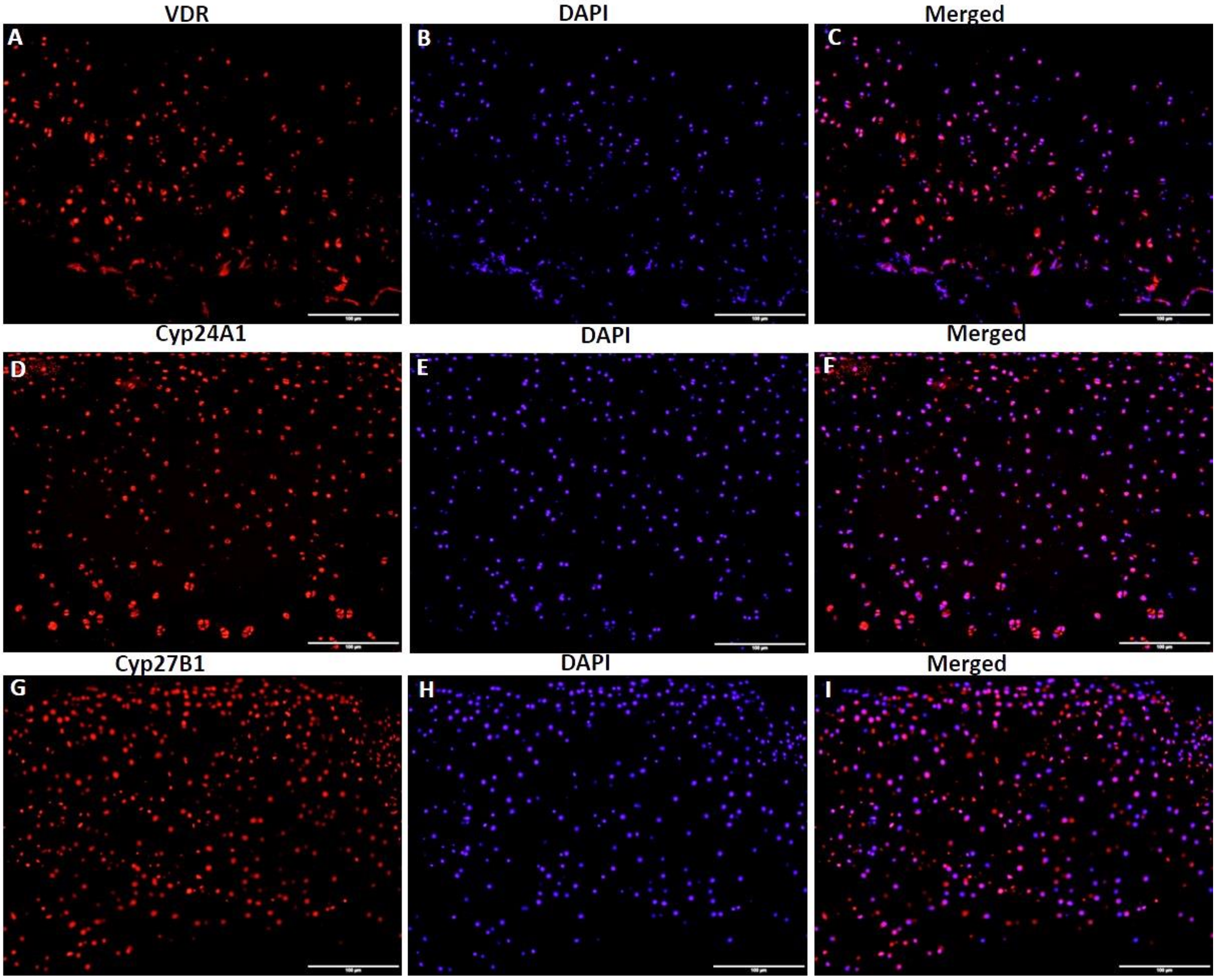

Figure 7. Immunofluorescence for VDR, Cyp24A1 and Cyp27B1 in VDDef, VDSuff, and VDSupp hyperlipidemic microswine articular cartilage. VDR- panel A, Cyp24A1- panel D, Cyp27B1- panel G, DAPI- panels B, E, and H, merged images- panels C, F, and I. DAPI- 2-4-amidinophenyl-1H -indole-6-carboxamidine, VDR- vitamin D receptor. These are the representative images from all the subjects in the study.

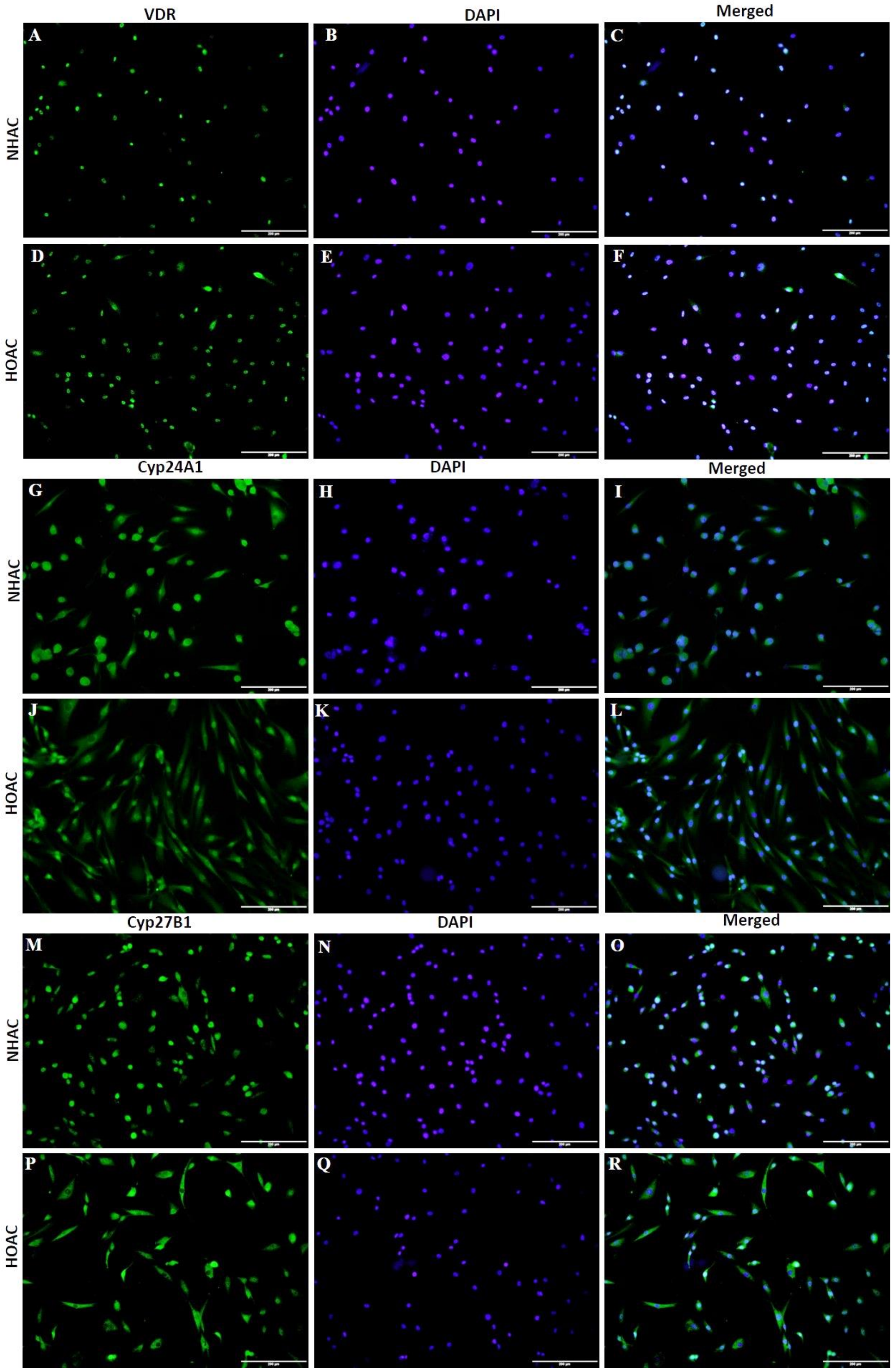

Figure S8. Immunofluorescence for VDR, Cyp24A1 and Cyp27B1 in NHAC and HCOA cells. VDR- panels A and D, Cyp24A1- panels G and J, Cyp27B1- panels M and P, DAPI- panels B, E, H, K, N, and Q, merged images- panels C, F, I, L, O, and R. DAPI- 2-4-amidinophenyl-1H -indole-6-carboxamidine, VDR- vitamin D receptor.
